# Comparative Metabolome and Transcriptome Analysis Reveals the Possible Roles of Rice Phospholipase A Genes in the Accumulation of Oil in Grains

**DOI:** 10.3390/ijms252111498

**Published:** 2024-10-26

**Authors:** Huasheng Cao, Rong Gong, Liang Xiong, Fujun Wang, Haiyong Gu, Shuguang Li, Gao He, Shihu Liang, Wenyong Luo, Xianjin Qiu

**Affiliations:** 1 Rice Research Institue, Guangdong Academy of Agricultural Sciences, Guangzhou 510640, China; caohuasheng@gdaas.cn (H.C.); gongrong@gdaas.cn (R.G.); xiongliang@gdaas.cn (L.X.); wangfujun@gdaas.cn (F.W.); guhaiyong@gdaas.cn (H.G.); hegao@gdaas.cn (G.H.); liangshihu@gdaas.cn (S.L.); 2 Key Laboratory of Genetics and Breeding of High Quality Rice in Southern China Co-Construction by Ministry, Ministry of Agriculture and Rural Affairs, Guangzhou 510640, China; 3 Guangdong Key Laboratory of New Technology in Rice Breeding, Guangzhou 510640, China; 4 Guangdong Rice Engineering Laboratory, Guangzhou 510640, China; 5 College of Agriculture, Yangte University, Jingzhou 434025, China

**Keywords:** rice grains, comparative metabolome and transcriptome, phospholipase A, oil quality

## Abstract

The phospholipase A (*PLA*) gene family plays a crucial role in the regulation of plant growth, development and stress response. Although *PLA* genes have been identified in various plant species, their specific functions and characteristics in oil quality formation of rice grains (*Oryza sativa* L.) have not been studied yet. Here, we identified and characterized 35 rice *PLA* genes, which were divided into three subgroups based on gene structures and phylogenetic relationships. These genes are distributed unevenly across 11 rice chromosomes. The promoter sequence of rice *PLAs* contain multiple plant hormones and stress-related elements. Gene expression analyses in various tissues and under stress conditions indicated that *PLAs* may be involved in rice growth, development and stress response. In addition, metabolomics, transcriptomics and qRT-PCR analyses between two rice varieties Guang8B (G8B, high oil content) and YueFengB (YFB, low oil content) revealed that the different expressional levels of rice *PLA* genes were closely related to the differences in the oil content between ‘G8B’ and ‘YFB’ grains. The findings of this study provide potential novel insights into the molecular information of the phospholipase A gene family in rice, and underscore the potential functions of *PLA* genes in rice oil content accumulation, providing valuable resources for future genetic improvement and breeding strategies.

## 1. Introduction

Rice, an important food crop only second to wheat in terms of sowing area and total yield, plays a pivotal position in world food production. It is the staple food for half of the world’s population (mainly China and India) and accounts for over 40% of the world’s food production [1]. Since the 1950s, through the unremitting efforts of several generations of breeders, the yield of rice has been greatly improved by dwarf breeding, hybrid breeding and other methods. With the refinement of dietary structures and the continuous improvement of living standards, people’s requirements for the appearance quality, palatability and nutritional quality of rice are constantly improving [2,3]. As one of the main storage substances in rice, lipids not only have rich nutritional value, but also have a significant impact on rice quality, particularly its cooking and eating quality [4,5,6].

Lipids in rice are mainly composed of triacylglycerols (TAGs), phospholipids (PLs) and free fatty acids (FFAs), which exist in the rice bran, embryo and endosperm [7,8]. Triglyceride is the main form in which lipids are stored in rice, generated by the dehydration and condensation of activated fatty acids and diacylglycerol [9]. They can rapidly accumulate in the grains of rice after 5–12 days of flowering [10,11]. Phospholipase A catalyzes the hydrolysis of various phospholipid components, such as phosphatidylcholine (PC), Phosphatidylglycerol (PG), Phosphatidylserine (PS) sn-1 or sn-2 acyl ester bonds, producing free fatty acids and corresponding lysophosphatides. It is one of the key enzymes in plant fatty acid and oil metabolism [12,13,14]. *PLA* genes have been isolated from a variety of plants, including Arabidopsis, rice, rapeseed, etc. [15,16,17,18,19], and are widely involved in a series of biological processes such as plant growth and development, biotic and abiotic stress responses [20,21,22]. Phospholipase A can be divided into phospholipase A1 (PLA1) and phospholipase A2 (PLA2) according to the different sites of action. PLA1 acts on the sn-1 acyl ester bond and can be divided into three subfamilies, PLA1I, PLA1II, and PLA1III, while PLA2 acts on the sn-2 acyl ester bond, including cytoplasmic phospholipase A2 (cPLA2), non Ca^2+^-dependent phospholipase A (iPLA2), secretory phospholipase A2 (sPLA2), platelet-activating factor acetylhydrolases (PAF-AHs) And lysosomal phospholipase A2 (PLA2s), both of which produce free fatty acids and lysophosphatids [23,24,25,26].

There are 12 *PLA1* genes in the Arabidopsis genome, among which *AtDAD1* is a *PLA1-Iβ* gene located in chloroplasts. Its product serves as a precursor for the initial synthesis of jasmonic acid and plays an important role in the maturation and cleavage of pollen outer walls [15]. Another member of the *PLA1-Iα* subfamily, *DGL*, is involved in wound responses mediated by methyl jasmonate and responses to pathogens [27]. Arabidopsis *sPLA2* is a kind of widely studied *PLA* gene. It is reported that different *sPLA2* genes have different subcellular localization and biological functions. AtsPLA2α is located in the apoplast and Golgi apparatus of mature leaves and is involved in auxin transport and signal transduction [28]. AtsPLA2β and AtsPLA2γ are both located in the endoplasmic reticulum and the former plays important roles in the stem orientation, stomata opening and pollen development, while the latter only participates in pollen germination and pollen tube elongation [29,30]. So far, no cPLA2- and iPLA2-homologous genes have been found in plant genomes, but there is a class of phospholipase A (patatin phospholipase A, pPLAs) with a higher homology to iPLA2 in plants [31]. In Arabidopsis, pPLA can be divided into three subfamilies (pPLAI, II and III), which are involved in disease resistance, growth and development, cellulose synthesis and double fertilization. Among them, the expression of *AtpPLAIIα* is induced by bacterial and fungal invasion, and its induction process depends on the jasmonic acid signaling pathway [32]. Overexpression of *AtpPLAIα* in Arabidopsis reduces plant resistance to gray mold, while down-regulation of *AtpPLAIIα* expression enhances resistance [33]. It has been reported that OspPLAIIIα plays an important role in the nutritional and reproductive growth of rice by mediating cellulose metabolism [17]. *DEP3* encodes a pPLAIIIδ, which participates in the regulation of spikelet morphology and grain size by regulating the development of vascular bundles [18]. *OsMATL* encodes pollen-specific phospholipase OspPLAIIβ, which is homologous to maize ZmMATL and induces haploid formation in rice [34].

In this study, we conducted a comprehensive examination of the *PLA* gene family in rice using the well-established IRGSP-1.0 reference genome (http://plants.ensembl.org/Oryza_sativa/Info Accessed on 20 July 2022) and successfully identified and characterized 35 *PLA* gene family members within the rice genome. This investigation provides new insights into the *PLA* genes through detailing the analysis of the gene structures, evolutionary relationships, expression profiles, metabolomics and transcriptomics in rice. Our results not only facilitate further research on *PLA* genes in rice but also contribute to the genetic improvement and breeding of high-quality-oil rice varieties.

## 2. Results

### 2.1. Basic Information of PLA Family Genes in Rice

In this study, we used two methods to identify the phospholipase A (*PLA*) genes in the rice genome. Initially, Arabidopsis PLA protein sequences were used to perform a BLASTP search against to the TIGR rice database (http://rice.uga.edu/index.shtml Accessed on 22 July 2022). Subsequently, the keyword “phospholipase A” was employed to search the NCBI (http://www.ncbi.nlm.nih.gov Accessed on 22 July 2022) and rice databases. By integrating the results from the searches above, 35 candidate *PLA* genes were obtained. According to our results, we can see that the rice PLA family can be divided into three subfamilies based on their conserved domains, including phospholipaseA1 (PLA1), patatin-related phospholipaseA (pPLA) and secretory phospholipase A2 (sPLA) which contain 12, 20 and 3 members respectively. Furthermore, the sequence analysis showed that the amino acids lengths of these PLA protein are ranging from 131 to 998. The predicted molecular weights of PLA proteins varied from 13.4 to 110 kDa. And the theoretical PI ranged from 5.07 to 11.73. The subcellular localization prediction results showed that these PLA proteins were widely localized in different organelles, including chloroplasts, nuclei, vacuoles and cell membrane, indicating their possible functional diversity in different organelles (Supplementary Table S1).

### 2.2. Phylogenetic Analysis of PLAs Between Arabidopsis and Rice

**Figure 1:**
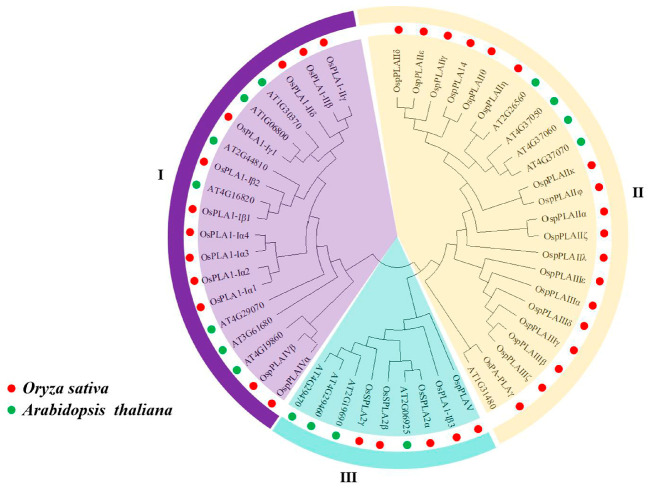
Phylogenetic analysis of PLAs between Arabidopsis and rice. The amino acids of Arabidopsis and rice PLAs were aligned and the phylogenetic tree was generated. The subgroups classified as I, II and III are represented and distinguished using different background colors. Red dots and green dots denote the PLAs from rice and Arabidopsis, respectively.

To better understand the evolutionary relationships and potential functions of the PLA family members, The phylogenetic analysis of the PLA proteins between rice and Arabidopsis was performed. Our results indicated that 50 PLAs formed three subfamilies (I, II and III), which is consistent with the search results above. The subfamily I contained twelve OsPLAs and seven AtPLAs. The subfamily II contained the highest number of PLAs, with eighteen OsPLAs and four AtPLAs. Interestingly, one subfamily I member, OsPLA1-Iβ3, and one subfamily II member, OspPLAV, showed a higher homology with subfamily III rather than subfamily I or II, respectively, which may be related to their short amino acid sequence. However, only seven members (OssPLA2α, OssPLA2β, OssPLA2γ, OsPLA1-Iβ3, OspPLAV, At2g06529 and At2g19690) were found in subfamily III (Figure 1).

### 2.3. Gene Structure and Motif Analysis

**Figure 2:**
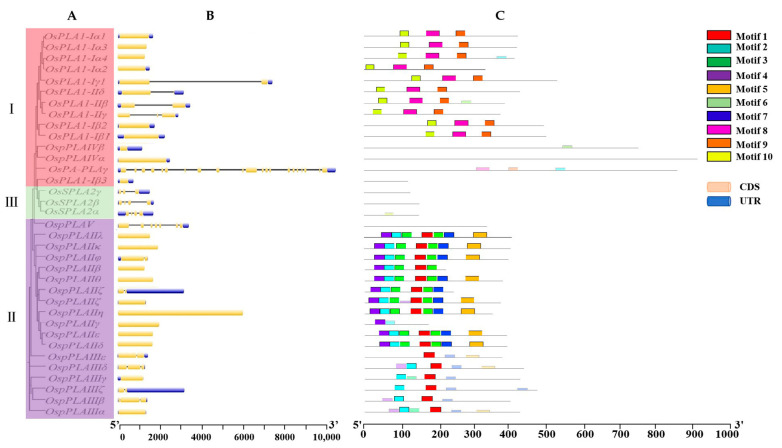
Phylogenetic relationships, gene structures and architecture of the conserved protein motifs in rice OsPLAs. (**A**) The phylogenetic tree was constructed based on the full-length sequences of rice PLA proteins, including OsPLA1 (I), OspPLA (II) and OssPLA2 (III). (**B**) Gene structures of *OsPLA* genes. The yellow box, blue box and gray line represent the exon, untranslated region (UTR) and intron, respectively. (**C**) Motif patterns of OsPLA proteins. Motifs numbered 1 to 10 are visually represented by distinct colored boxes. Detailed sequence information for each motif can be found in Supplementary Table S2.

Based on gDNA and CDS sequences, the gene structures of *OsPLA* genes were analyzed, including untranslated regions (UTRs), exons and introns. With the exception of *OsPA-PLAγ*, which contains twenty-one exons, the remaining 11 members of subfamily I possess one to three exons. Similarities were observed in the subfamily II, 19 members, excluding *OspPLAV*, contain less than three exons. However, in subfamily III, all the *PLA* genes contain three to four exons, evenly two times more than that in the members of subfamilies I and II, indicating a notable difference (Figure 2A,B). Additionally, fifteen motifs were found within the 31 OsPLA proteins according to the motif analysis. Motifs 8, 9, 10 and 11 were found in all members except OsPLA1-Iβ3 and OsPA-PLAγ in subfamily I. However, according to the motif number, the members of subfamily II can be broadly divided into two categories; the first one includes OsPLAIIs members, most of which contain motifs 1, 2, 3, 4, 6 and 15. The other includes OsPLAIIIs members, which possess motifs 1, 2, 5 and 6. To our surprise, no motifs were found in the members of subfamily III, indicating a functional differentiation among the members of the OsPLA family (Figure 2C).

### 2.4. Chromosomal Distribution and Gene Duplication Events of OsPLAs

**Figure 3:**
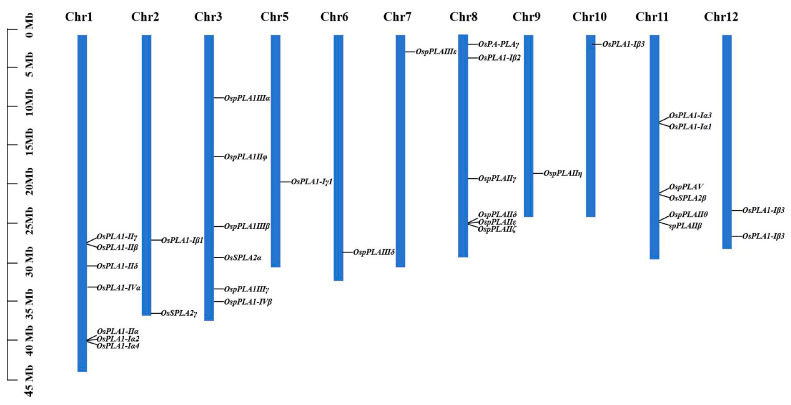
Distribution of the *PLA* genes on the 12 chromosomes of rice. The black lines on the right side of the chromosomes denote the location of rice *PLA* genes.

**Figure 4:**
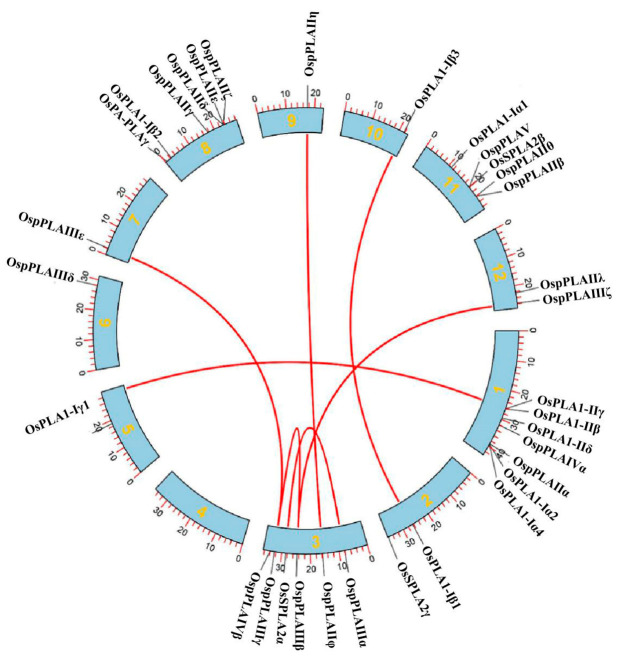
Schematic presentations of the inter-chromosomal and intra-chromosomal relationships of rice *PLA* genes. The red lines indicate duplicated *PLA* gene pairs in rice. The chromosome numbers are indicated in the middle of each chromosome.

In this study, we mapped 35 *OsPLAs* onto 12 chromosomes of rice. The distribution of *OsPLA* gene family members on each chromosome ranged from one to seven, except for chromosome 4 which contained no *PLA* gene distribution. Chromosome 5, 6, 7, 9 and 10 only possessed one *OsPLA* gene, while chromosome 1 contained seven *OsPLA* genes (Figure 3). It is well known that gene replication is the source of genetic innovation and evolutionary innovation. It is a rapid mechanism that generates additional sequences for natural selection to improve the adaptability of organisms. To better study the evolution of *OsPLA* genes, the McScan X software was used to explore genome-wide replication events. In our study, we identified that 40% of *OsPLAs* present duplication events, including two intra-chromosomal duplications in Chr3 and five inter-chromosomal duplications in Chr1-Chr5, Chr2-Chr10, Chr3-Chr7, Chr3-Chr9 and Chr3-Chr12. The results above suggest that these *PLA* genes might have been generated through genome-wide duplication events, which highlighted the importance of such events in the expansion of rice *OsPLA* gene family (Figure 4).

### 2.5. Cis-Acting Regulatory Elements in OsPLAs Promoters

**Figure 5:**
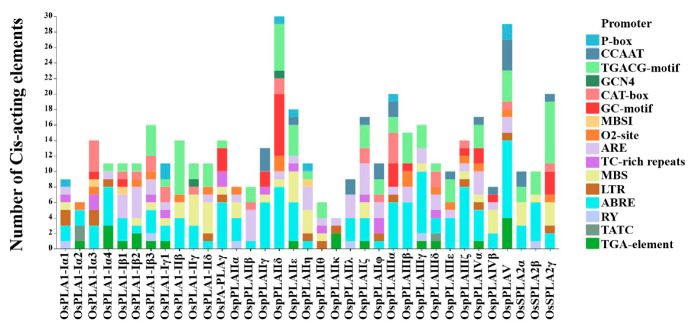
Cis-acting element analysis of *OsPLA* genes in rice. The X axis indicates the rice *PLA* genes, and the Y axis indicates the number of cis-acting elements in each *OsPLA*. Different colored squares represent distinct cis-acting elements.

The cis-acting elements on the upstream 2 Kb sequence of the starting codon of all 35 *OsPLA* genes were predicted using the Plant CARE online analysis website (http://bioinformatics.psb.ugent.be/webtools/plantcare/html/ Accessed on 20 October 2022). Our results indicated that there are 15 cis-acting elements related to plant hormones and abiotic stress in the promoter sequences of *OsPLA* family members, including P-box and TATC element (gibberellin signal response element), ABRE element (abscisic acid signal response element), the TGA element (auxin signal element), TGACG motif (methyl jasmonate response element), GC motif (hypoxia-related element), ARE (anaerobic regulatory element), TC-rich repeats (pressure response related), MBSI (drought-related element) and LTR (low temperature response) element. Further analysis showed that almost all rice *PLA* members contain ABRE regulatory elements in their promoter regions, thirteen members contain TGA elements, and seven members contain P-box elements, indicating that these members may be widely involved in plant responses to different hormones and display functional redundancy. However, MBSI and GCN4 (endosperm-specific expressional element) only exist in the promoter regions of *OsPLA1-Iα3*, *OsPLA1-Iβ1*, *OspPLAIIη* and *OsPLA1-IIγ*, *OspPLAIIδ,* respectively, meaning that there are functional differentiation between *OsPLA* family members (Figure 5).

### 2.6. Expression Patterns of OsPLAs in Different Rice Tissues and Plant Hormones Response

**Figure 6:**
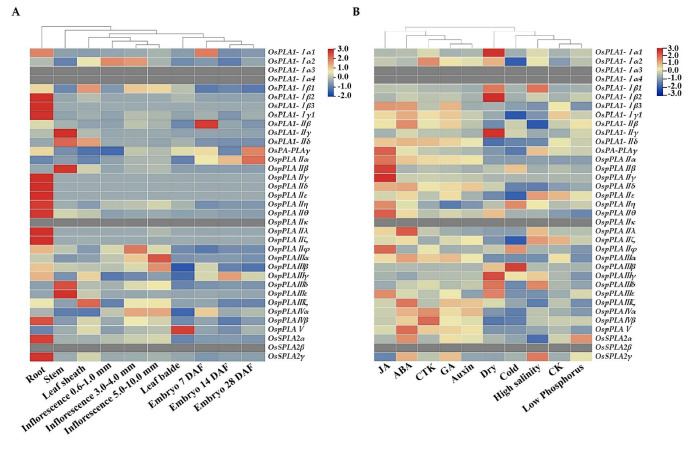
(**A**) Expression profiles of rice *PLA* genes in different developmental stages. The transcriptomic data were normalized via log2 to generate a heatmap. (**B**) Expression profiles of *PLA* genes in rice under plant hormones and different abiotic stresses. The transcriptomic data were normalized via log2 to generate a heatmap.

To explore the potential functions of the *OsPLA* genes in different developmental stages of rice organs/tissues, various plant hormone responses and abiotic stress, RNA-seq data from NCBI database (http://www.ncbi.nlm.nih.gov Accessed on 20 September 2023) were used to analyze their expression patterns. The heat map results showed the significantly different expression patterns of *OsPLA* family members in different rice tissues (Figure 6A). Four genes, *OsPLA1-Iα3*, *OsPLA1-Iα4*, *OspPLAIIκ* and *OspPLAIVβ* could not be detected in any tissues, meaning that they are all pseudogenes or needed to been induced by special conditions. Further studies showed that about one-third of the *OsPLAs* (*OsPLA1-Iβ2*, *OsPLA1-Iβ3*, *OsPLA1-Iγ1*, *OspPLAIIγ*, *OspPLAIIδ*, *OspPLAIIε*, *OspPLAIIθ*, *OspPLAIIλ*, *OspPLAIIζ* and *OspPLAIVβ*) can only been detected in root tissue, indicating their important roles in the growth and development of the rice root system. However, *OsPLA1-IIγ*, *OsPLA1-IIδ*, *OspPLAII*β, *OspPLAIIIδ* and *OspPLAIIIε* showed the highest expression levels in the rice stem tissue. In addition, our results indicated that only several *OsPLA* genes can been detected in rice seed development stage. For example, the expression of *OsPLA1-Iα1*, *OsPLA1-IIβ* and *OsPA-PLAγ* can be monitored in the early stage. (7 days after flowering, DAF), whereas *OspPLAIIα* and *OspPLAIIIγ* showed the high expression levels during the early (embryo 07 DAF), middle (14 days after flowering, DAF) and later stages (28 days after flowering, DAF) of seed development.

To identify whether the *OsPLA* genes are involved in plant hormones and abiotic stress responses, we used RNA-seq data to analyze their expression levels under Jasmonate, Abscisic acid, Cytokinin, Gibberellin, Auxin, dry, cold, high-salinity and low-phosphorus treatments. Except for possible pseudogenes, about one-third of the *PLA* genes (*OsPLA1-Iβ3*, *OsPA-PLAγ*, *OspPLAIIα*, *OspPLAIIβ*, *OspPLAIIγ*, *OspPLAIIη*, *OspPLAIIθ*, *OspPLAIIφ* and *OspPLAIIIε*) are strongly induced by JA, which is closely related to their functions in JA-mediated disease resistance responses. In addition, ABA can also strongly induce the expression of *OsPLA* genes (*OsPLA1-Iβ3*, *OsPLA1-IIβ*, *OspPLAIIλ*, *OspPLAIIIα*, *OspPLAIIIζ*, *OspPLAIVα*, *OspPLAIVβ*, *OspPLAV*, *OsSPLA2α* and *OsSPLA2γ*), indicating that these *OsPLAs* may play important roles in ABA signal transduction. In terms of abiotic stress response, *OsPLA1-Iα1*, *OsPLA1-Iβ1*, *OsPLA1-Iβ2*, *OsPLA1-IIγ*, *OspPLAIIIγ* and *OspPLAIIIε* were specifically induced by drought stress, while *OspPLAIIIβ* showed a significant increase in expression levels under cold stress conditions. The results above fully demonstrate the functional diversity of the *OsPLA* gene family members in rice (Figure 6B).

### 2.7. Metabolomics and Transcriptomics Analysis of the Roles of OsPLAs in Rice Oil Accumulation

**Figure 7:**
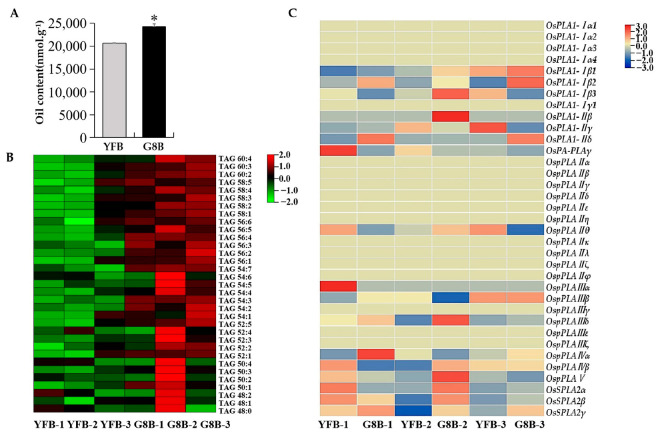
Oil content and rice *PLA* genes expressional analyses in grains of rice varieties ‘YueFeng B’ and ‘Guang 8B’. (**A**) Determinant of total oil (triacylglycerol, TAG) content in ‘YueFeng B’ and ‘Guang 8B’ seeds at stage III (30 DAF). * represents a significant difference at the *p* < 0.05 level. (**B**) Analysis of oil component contents in ‘YueFeng B (YFB)’ and ‘Guang 8B (G8B)’ grains at stage III (30 DAF). The metabolomics data were normalized via log2 to generate a heatmap. YFB-1/2/3 and G8B-1/2/3 denote biological repeats of YFB and G8B, respectively. (**C**) Expression analysis of *PLA* genes in rice grains at different development stages (10 DAF, 20 DAF and 30 DAF). The transcriptomics data were normalized via log2 to generate a heatmap. YFB-1/2/3 and G8B-1/2/3 denote the samples in stages I, II and III (10/20/30 DAF) of YFB and G8B, respectively.

To gain a thorough understanding of the role of *OsPLAs* genes in rice seed oil synthesis, the oil contents of the rice varieties ‘YueFeng B’ (YFB) and ‘Guang 8B’ (G8B) at 30 days after flowering were analyzed. The results showed that the oil content in the ‘G8B’ grains was significantly higher than that in ‘YFB’ (Figure 7A). The analysis of the TAG components from the different rice varieties showed that almost all of the TAG components in ‘G8B’ were higher than those in ‘YFB’, especially the TAGs containing rich unsaturated fatty acids, such as TAG (50:2), TAG (50:3), TAG (50:4), TAG (52:3), TAG (52:4), TAG (54:4), TAG (54:5), TAG (54:6), TAG (56:5), TAG (58:4) and TAG (60:4), indicating the better nutritional quality of ‘G8B’ grains compared to ‘YFB’ grains (Figure 7B).

Furthermore, a comparative transcriptomic method was used and the expression levels of *OsPLA* genes in the different developmental stages of rice seeds (10 DAF, 20 DAF, 30 DAF) between the ‘YFB’ and ‘G8B’ grains were analyzed. Our results indicated that, in developmental stage I (10 DAF), the expression levels of *OsPLA1-IIβ*, *OsPLA1-IIβ*, *OspPLAIIIδ* and *OspPLAV* in ‘G8B’ grains were higher than those in ‘YFB’, whereas the expression levels of *OspPLAIIIα* and *OsPA-PLAγ*, which can hydrolyze phosphatidic acid to generate lysophosphatidic acid, were lower than those in ‘YFB’. In developmental stage II (20 DAF), the expression levels of *OspPLAIIγ* and *OspPLAIIIβ* in the ‘G8B’ grains were higher than that in the ‘YFB’; on the contrary, *OspPLAIVα*, *OsSPLA2β* and *OsSPLA2γ* showed the opposite trend. In developmental stage III (30 DAF), we was also found that three *PLAI* subfamily members (*OsPLA1-Iβ1*, *OsPLA1-Iβ2*, *OsPLA1-IIδ*), one *PLAII* subfamily member, *OspPLAIIIβ*, and most of the *PLAIII* members, *OsSPLA2β* and *OsSPLA2γ*, exhibited higher expression in the ‘G8B’ grain samples than that in the ‘YFB’, but only *OsPLA1-IIγ* belonging to the *PLAI* subfamily showed alower expression level (Figure 7C).

### 2.8. Expression Analysis of OsPLAs in Rice Grains by qRT-PCR

**Figure 8:**
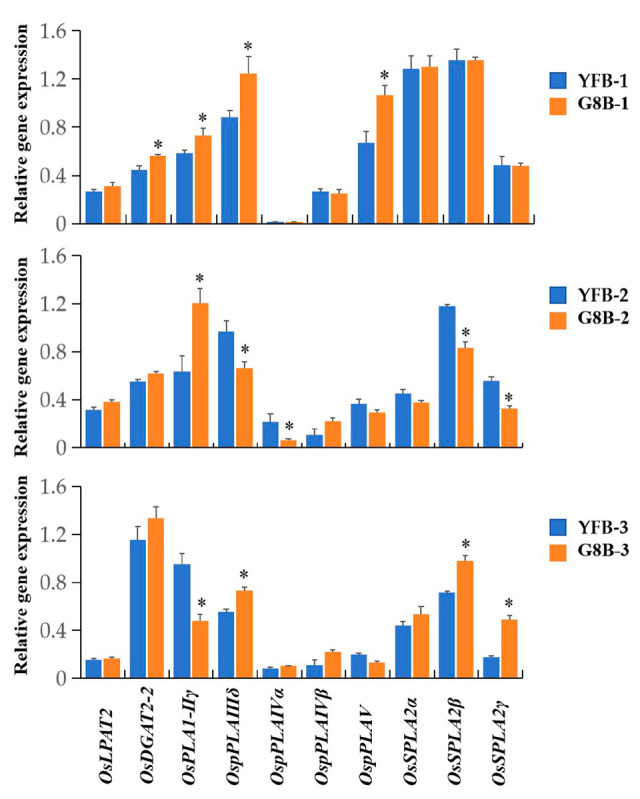
Analysis of the *OsPLAs’* expression profiles at different matural stages of rice grain. The relative expressions of *OsPLAs* were analyzed by qRT-PCR. The mean values and standard errors (SE) were calculated from three biological replicates, each consisting of three technical replicates. * *p* < 0.05 (Student’s *t*-test).

To verify the transcriptome data, qRT-PCR was used to analyze the expression levels of *OsPLA* genes at different developmental stages of rice grains. As shown in Figure 8, the qRT-PCR data are generally consistent with the transcriptome analysis results. Specifically, in stage I, the expression levels of *OsDGAT2-2*, *OsPLA1-IIγ*, *OspPLA IIIδ* and *OspPLAV* in ‘G8B’ were significantly higher than those in ‘YFB’. However, in Stage II, only *OsPLA1-IIγ* was significantly higher in ‘G8B’ than that in ‘YFB’. On the contrary, the expression levels of *OspPLAIIIδ*, *OsSPLA2β* and *OsSPLA2γ* were significantly lower in ‘G8B’ than those in ‘YFB’. Our results also showed that, in stage III, the expression levels of *OsSPLA2β* and *OspPLAIIIδ* in ‘G8B’ were significantly higher than those in ‘YFB’, contrary to the results in stage II, However, a significantly lower expression of *OsPLA1-IIγ* and *OsSPLA2γ* were also observed. According to the results above, we suggest that the significantly higher expression levels of *OsPLA1-IIγ*, *OspPLAIIIδ*, *OspPLAV*, *OsSPLA2β* and *OsSPLA2γ* in ‘G8B’ compared to those in ‘YFB’ might be an important reason for the higher oil content in ‘G8B’ than in ‘YFB’.

## 3. Discussion

Phospholipase A (PLA; EC 3.1.1.32) catalyzes the hydrolysis of various phospholipid components sn-1 or sn-2 acyl ester bonds, to produce free fatty acids and corresponding lysophosphatides. Increasing evidence suggests that PLAs are widely involved in plant growth, development and stress response [20,21,22]. In this study, 35 OsPLA members were identified and characterized based on the rice reference genome. All identified OsPLAs can be divided into three distinct clusters (termed subfamilies I, II, and III) on the basis of their conserve domain and phylogenetic analysis. Our analysis showed that the conserved domain and motifs in OsPLAs were relatively conserved within the same cluster (Figure 1 and Figure 2). Additionally, the subcellular location prediction results indicated that most of the OsPLAs are located in the chloroplast, vacuole and cell membrane, which still needed to be further validation. Gene replication events are the basis of gene family expansion, which can promote organisms to adapt to different environmental conditions [35]. Our results revealed two intra-chromosomal duplication events and five inter-chromosomal duplication events in the *OsPLA* gene family, suggesting the importance of such events in the expansion of *OsPLAs*’s (Figure 4). The *OsPLA* gene family members are widely expressed across different tissues. The RNA-seq analysis revealed that about one-third of the *OsPLAs* exhibited the highest expression levels in the root, indicating their potential involvement in root growth and development (Figure 6A). The *OsPLAs* promoter analysis identified a series of cis-acting elements, including those elements related to development, environmental stress and hormone responsiveness (Figure 5), which suggests *OsPLAs* may play important roles in rice’s responses to plant hormones, abiotic and biotic stresses. To further verify this hypothesis, the expression of *OsPLAs* under different plant hormones and abiotic stresses (NaCl, cold, drought, low phosphorus treatments) were explored. As shown in Figure 6B, the results showed that the expressional levels of most of the *OsPLAs* were significantly influenced by plant hormones or abiotic stress. These outcomes verify the hypothesis derived from the promoter analysis and suggest that the *OsPLAs* have diverse functions and widely participate in the response to plant hormones and abiotic stress.

Triacylglycerol (TAG) and their derivatives are not only important energy storage substances, but are also indispensable components of biopolymers such as cutin in plants [36]. In addition, the important roles of TAGs in seed germination and plant sexual reproduction were also confirmed [37,38]. It was reported that phospholipase A is one of the key enzymes in oil synthesis, and plays an important role in increasing seed oil content and improving seed oil quality [12]. In this study, we found that the rice PLA family members play roles in the different stages of grain development. At the early stage of grain development (10 DAF), the expression levels of *OsPLA1-IIγ*, *OspPLAIIIδ* and *OspPLAV* in the high oil content rice grain (G8B) were significantly higher than those in the low oil content rice grain (YFB). At the middle stage (20 DAF), the expression level of another phospholipase A, *OsPLA1-IIγ*, in the ‘G8B‘ grains was significantly higher than that in YFB. And in the later stage (30 DAF), significantly different expressions of *OspPLAIIIδ*, *OsSPLA2β* and *OsSPLA2γ* were found between ‘G8B‘ and ‘YFB’. Surprisingly, we also observed that the expression levels of *OspPLAIIIδ*, *OspPLAIVα*, *OsSPLA2β*, *OsSPLA2γ*, and *sPLA1-IIγ* in GB8 were significantly lower than those in YFB in the middle and late stages, respectively. Phospholipase A not only catalyzes the hydrolysis of phosphatidylcholine (PC), phosphatidylglycerol (PE), phosphatidylserine (PS), phosphatidylethanolamine (PE) and phosphatidylinositol (PI), but also uses phosphatidic acid (PA) as a substrate to produce free fatty acids and lysophosphatidic acid [12]. In this study, the lower expression levels of *OsPLAs* in GB8 compared to YFB reveal their potential phosphatidic acid degradation activity in rice. PA is an important intermediate product [37,38] of oil synthesis, and its reduction will greatly affect the synthesis of TAGs [39,40]. Therefore, we speculate that *OspPLAIIIδ*, *OspPLAIVα*, *OsSPLA2β*, *OsSPLA2γ* and *sPLA1-IIγ* may use phosphatidic acid as a substrate to produce lysophosphatidic acid, and their reduced expression levels are also beneficial for rice oil synthesis.

## 4. Materials and Methods

### 4.1. Plant Materials and Treatments

Rice (*Oryza sativa* L.) plants of two indica varieties, Guang8B and YuefengB (supplied by the Rice Research Institute, Guangdong Academy of Agricultural Sciences, Guangzhou, China), were used in this study. Guang8B is the maintainer line of Guang8A, the first silk-seedling sterile line in Guangdong Province, with the characteristics of a good taste and sufficient oil content. These two indica rice materials were grown in Guangzhou, Guangdong Province (eastern longitude 112°57′~114°3′; northern latitude 22°26′~23°56′). The rice seed samples were obtained 10, 20 and 30 days after flowering, respectively, and then the transcriptome and lipid metabolome tests were performed to analyze the oil content and the expression levels of *OsPLAs* in the different seed development stages between Guang8B (G8B) and YuefengB (YFB).

### 4.2. Genome-Wide Identification and Annotation of PLA Genes in Rice

The protein sequences of the *PLA* in Arabidopsis were obtained from the Arabidopsis Information Resource (TAIR) database (http://www.arabidopsis.org Accessed on 20 September 2023). These proteins were aligned, and the output was used to build a Hidden Markov Model (HMM), which was used to search for homologous proteins in rice using HMMER 3.3.2. The genomic assembly of rice was downloaded from NCBI (https://www.ncbi.nlm.nih.gov Accessed on 20 September 2023). The putative PLA conserved protein domains were verified by utilizing the Conserved Domain Database (CDD) (https://www.ncbi.nlm.nih.gov/cdd Accessed on 20 September 2023). Data regarding the isoelectric point (IP), grand average of hydropathicity, instability index, aliphatic index and relative molecular mass of the OsPLA proteins were acquired from the website (https://web.expasy.org/compute_pi/ Accessed on 25 September 2023). The subcellular localization of OsPLA proteins was predicted using the Plant-mSubP online tool (https://bioinfo.usu.edu/Plant-mSubP/ Accessed on 25 September 2023).

### 4.3. Phylogenetic Analysis

To align the full-length protein sequences of PLAs in Arabidopsis and rice, MAFFT was utilized with the default parameters. The alignment results were then used to construct a neighbor-joining (NJ) tree using MEGA5. The parameters for constructing the NJ tree were set as follows: Poisson model, pairwise deletion and 1000 replicates for bootstrap values.

### 4.4. Genetic Structure, Motifs Composition, Cis-Acting Elements and Gene Duplication

The exon–intron structure of the *OsPLA* genes was determined using the Gene Structure Display Server (GSDS: http://gsds.cbi.pku.edu.cn/ Accessed on 2 October 2023). To identify conserved motifs within the OsPLA proteins, we employed the MEME online tool available at (http://meme-suite.org/tools/meme Accessed on 2 October 2023). Furthermore, the plantCARE website (https://www.plantcare.co.uk/ Accessed on 2 October 2023) was utilized to predict the cis-acting elements within the promoters of *OsPLAs*. An analysis of gene duplication events in rice *PLA* members was conducted using the multiple collinear scanning toolkit (MCScanX).

### 4.5. Chromosomal Localization

The genome and annotation files of the rice were obtained from Ensemble Plants (http://plants.ensembl.org/index.html Accessed on 8 October 2023), and then gene position, gene collinearity and chromosome length files were extracted and the results were visualized using TBtools.

### 4.6. Transcriptome Expression Pattern of OsPLAs

The RAP-DB (https://ricexpro.dna.affrc.go.jp/ Accessed on 8 October 2023) database was used to analyze the spatial and temporal expression profiles of *OsPLAs*. First, the expression levels of *OsPLAs* in root, stem, leaf sheath, leaves, inflorescences of 0.6–1 mm, 3.0–4.0 mm, 5.0–10 mm length and embryos at 7, 14 and 28 days were obtained, and the results were visualized using the TBtools software (v1.108).

### 4.7. OsPLA Gene Expression Analysis by qRT–PCR

To understand the role of *OsPLA* genes in the oil accumulation in ‘G8B’ and ‘YFB’, we conducted a qRT-PCR analysis. The primers were designed according to the CDSs by Primer 5.0 software. All primers were verified by qRT–PCR, and the list of primers used is shown in Supplementary Table S3. Primers were produced by Shanghai Biotechnology Co., Ltd. (Shanghai, China). The ACTIN gene was used as the internal reference. qRT-PCR was carried out on the Roche Light Cycler 480 II platform. Data analysis was performed with Excel 2013, graphs were drawn by R 4.2.2 software, and significance was determined with ANOVA in the R 4.2.2 software; *p* < 0.05 was considered significant. Relative gene expression was calculated using the 2^−∆∆Ct^ method.

## 5. Conclusions

So far, there are few reports on the function of the PLA gene family in rice [41,42,43], and the role of this family members in rice oil accumulation has not been reported yet. In this study, using a blend of genomic, bioinformatic and experimental approaches, a total of 35 *OsPLA* genes were identified in rice genome, and they exhibited an uneven distribution across twelve chromosomes. Based on the conserved domains and phylogenetic analysis, the OsPLAs were classified into three groups. Furthermore, our analysis indicated that 12 *OsPLA* genes likely originated from duplication events. Additionally, the promoter analysis revealed that hormones and stress response elements are widely presented in the *OsPLAs’* gene promoters, suggesting their important roles in hormone and stress responses. Moreover, our results suggest that the different expression levels of *OsPLAs* between ‘G8B‘ and ‘YFB‘ might be an important reason for the different oil contents in these two rice varieties. Overall, the study provides a scientific basis for understanding the function of OsPLAs in stress responses and the oil accumulation in rice grains, which will facilitate cloning and analyzing the function of *OsPLA* genes in the future.

## Data Availability

All the data created or analyzed for this investigation are presented in this publication.

## Supplementary Materials

The supporting information can be downloaded at: https://www.mdpi.com/article/10.3390/ijms252111498/s1.
