# Supplementary material for: Comparative Metabolome and Transcriptome Analysis Reveals the Possible Roles of Rice Phospholipase A Genes in the Accumulation of Oil in Grains
Source: Int J Mol Sci. 2024 Oct 26;25(21):11498. doi: 10.3390/ijms252111498 (PMC11546879; doi:10.3390/ijms252111498)
Supplement: Supplementary file 1 [file ijms-25-11498-s001.zip › Supplementary table S1.pdf]

Supplementary table S1. List of the 35 *OsPLA* genes identified in this study.

Locus_ ID	Gene Name	Protein Length	Molecular Weight (Da)	Theoretical PI	Instability Index	Aliphatic Index	Grand Average of Hydropathicity	Subcellular Location
OsPLA1(I)								
Os011g0299300	*OsPLA1-Iα1*	460	49800.09	8.62	35.78	85.91	-0.099	Chloroplast
Os01g0900400	*OsPLA1-Iα2*	362	38729.33	8.18	36.39	86.8	-0.018	Chloroplast
Os11g0298800	*OsPLA1-Iα3*	457	48670.53	8.86	38.59	85.49	-0.001	Chloroplast, Peroxisome
Os01g0900700	*OsPLA1-Iα4*	450	47098.41	8.1	41.83	79.89	-0.032	Chloroplast、Nucleus
Os02g0653900	*OsPLA1-Iβ1*	544	58922.97	9.61	57.96	80.4	-0.397	Chloroplast、Nucleus
Os08g0143600	*OsPLA1-Iβ2*	538	57567.76	9.08	54.66	85.54	-0.168	Chloroplast、Nucleus
Os10g0552200	*OsPLA1-Iβ3*	131	13381.85	7.5	30.82	117.63	0.639	Cell Membrane
Os05g0390000	*OsPLA1-Iγ1*	577	64524.16	6.35	47.58	73.97	-0.523	Chloroplast、Nucleus
Os01g0651800	*OsPLA1-IIβ*	420	45328.28	7.67	38.98	87.86	-0.123	Mitochondrion、Peroxisome
Os01g0651200	*OsPLA1-IIγ*	408	45619.03	5.59	45.74	77.72	-0.468	Chloroplast
Os01g0710700	*OsPLA1-IIδ*	465	50453.18	6.52	37.64	86.58	-0.218	Cell Wall、Nucleus
Os08g0110700	*OsPA-PLAγ*	937	104901.36	5.57	47.05	75.43	-0.539	Chloroplast
OspPLA(II)								
Os01g0898500	*OspPLAIIα*	411	45577	8.29	42.73	93.09	-0.101	Vacuole
Os11g0614500	*OspPLAIIβ*	245	26307.93	5.07	35.77	92.45	0.002	Cell Membrane、Vacuole
Os08g0376500	*OspPLAIIγ*	442	46774.36	6.98	44.39	92.13	-0.043	Vacuole
Os08g0476900	*OspPLAIIδ*	430	46813.66	7.63	35.58	90.02	-0.203	Vacuole
Os08g0477100	*OspPLAIIε*	431	46752.44	6.67	36.17	88.7	-0.208	Cell Membrane、Vacuole
Os09g0462400	*OspPLAIIη*	387	42647.13	7.15	39.34	92.25	-0.12	Chloroplast、Vacuole
Os11g0614400	*OspPLAIIθ*	417	45256.63	5.78	40.07	88.94	-0.12	Vacuole
Os12g0551600	*OspPLAIIκ*	437	48072.13	6.29	40.96	86.68	-0.141	Chloroplast、Vacuole
Os12g0552200	*OspPLAIIλ*	193	19698.32	11.73	88.28	53.21	-0.382	Chloroplast、Nucleus
Os08g0477500	*OspPLAIIζ*	269	29461.18	9.25	39.1	93.57	0.001	Vacuole
Os03g0393900	*OspPLAIIφ*	432	46400.23	8.78	38.41	82.75	-0.113	Chloroplast、Vacuole
Os03g0254400	*OspPLAIIIα*	469	49701.33	6.21	39.31	79.06	-0.143	Chloroplast、Vacuole
Os03g0640000	*OspPLAIIIβ*	441	45147.95	9.23	44.34	74.81	0.003	Vacuole
Os03g0784100	*OspPLAIIIγ*	470	48398.77	6.98	39.03	81.96	-0.016	Vacuole
Os06g0677000	*OspPLAIIIδ*	481	50265.29	10.01	42.62	79.69	-0.036	Chloroplast、Vacuole
Os07g0144500	*OspPLAIIIε*	416	43047.84	11.12	71.94	62.67	-0.244	Vacuole
Os12g0611300	*OspPLAIIIζ*	522	53001.78	9.62	50.67	77.95	-0.061	Vacuole
Os01g0762000	*OspPLAIVα*	998	110065.74	6.07	49.36	87.01	-0.264	Chloroplast、Vacuole
Os03g0810900	*OspPLAIVβ*	820	90395.52	6.53	47.98	85.4	-0.248	Vacuole
Os11g0546300	*OspPLAV*	367	39619.15	6.25	43.47	88.86	-0.147	Chloroplast、Vacuole
OssPLA2(III)								
Os03g0708000	*OsSPLA2α*	163	17113.75	5.76	60.46	95.89	0.225	Cell Membrane、Chloroplast、
Os11g0546600	*OsSPLA2β*	164	17305.66	4.91	43.42	78.78	0.04	Cell Membrane、Nucleus
Os02g0831700	*OsSPLA2γ*	138	14884.5	8.45	50.66	65.07	-0.149	Golgi
