# Supplementary material for: Comparative Metabolome and Transcriptome Analysis Reveals the Possible Roles of Rice Phospholipase A Genes in the Accumulation of Oil in Grains
Source: Int J Mol Sci. 2024 Oct 26;25(21):11498. doi: 10.3390/ijms252111498 (PMC11546879; doi:10.3390/ijms252111498)
Supplement: Supplementary file 1 [file ijms-25-11498-s001.zip › Supplementary table S2.pdf]

Supplementary table S2. Amino acid sequence of conserved motif consensus.

Motif	Motif Consensus
Motif 1	GDTTLRDTVTNVVIPTFDIKLLQPTIFSRYDAK
Motif 2	QELDGPDARIADYFDVIAGTSTGGLITAM
Motif 3	LKNALLSDICISTSAAPTYLPAHFFETTD
Motif 4	YGSLVTVLSIDGGGIRGIIPGTILAFLEEKL
Motif 5	PLIDIFTQASADLVDIHASVLFQALHCEKNYLRIQDDELTG
Motif 6	APBDNGRPLFAAKDINDFYLEHGPKIFPQ
Motif 7	GKPREFNLIDGGVAANNPTLVAMTHISKQI
Motif 8	AVATDEEAARLGRRDIVVAFRGTVTALEWVANLMSSLVPA
Motif 9	YKGEDVSITVTGHSLGAALALLLAYDJA
Motif 10	DPDLRREIIRYGELVQATYDAFDSDP
