# Supplementary material for: Comparative Metabolome and Transcriptome Analysis Reveals the Possible Roles of Rice Phospholipase A Genes in the Accumulation of Oil in Grains
Source: Int J Mol Sci. 2024 Oct 26;25(21):11498. doi: 10.3390/ijms252111498 (PMC11546879; doi:10.3390/ijms252111498)
Supplement: Supplementary file 1 [file ijms-25-11498-s001.zip › Supplementary table S3.pdf]

Supplementary table S3. The primer sequences for qRT-PCR used in this study

Primer	Sequence
LPAT2F	AAGGCAATGGGGAATGAGCA
LPAT2R	TGCCCAGCTCCTTTCCAAAA
DGAT2F	TGAGCCGCATTCTGTTTTGC
DGAT2R	CTGGCACTATGACGCAGCTA
OsPLA1-IIγF	TGTACACCTCGGAGGACCAA
OsPLA1-IIγR	TGAGGCTGGACGTGTTGTAC
OspPLAIIIδF	AGTCCAACTTCCTCTTCGGC
OspPLAIIIδR	CAGGTCAAAGTAGTCGGCGA
OspPLAIVαF	AAGGAAGCATGAGCCACCTC
OspPLAIVαR	TCCAAGCTCCCATCTCTCCA
OspPLAIVβF	CTCTCCACTCCTCCGACTGA
OspPLAIVβR	TGCAGCTCCGCATATGTAGG
OspPLAVF	GCTCTTCCCCTACCACATCG
OspPLAVR	CCTTGAGTACAGCCCCAAGG
OsSPLA2αF	GTACTGCGGCATCCTGTACA
OsSPLA2αR	AGCGAGATCACGTCGATGAC
OsSPLA2βF	GCCGACAACGACGAGAAGT
OsSPLA2βR	AGGTAGTCATTGTCGGTGGC
OsSPLA2γF	AGGGATCCGGTATGGCAAGT
OsSPLA2γR	TCCCTGAGGTCATTGTTGCC
